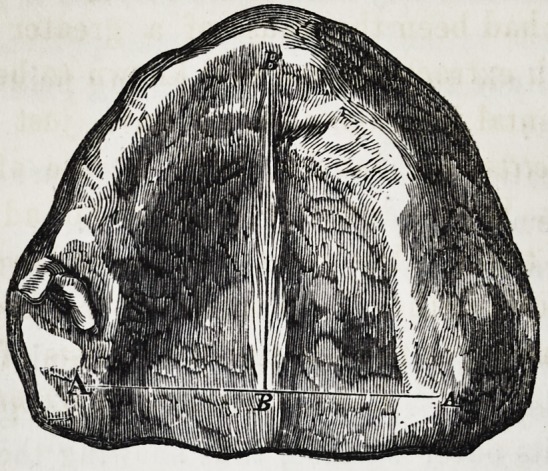# Case in Which the Upper Jaw Was Entirely Free from Teeth at Nine Years of Age

**Published:** 1857-07

**Authors:** Samuel Parker

**Affiliations:** Birmingham.


### ARTICLE XIII

### *Case in which the Upper Jaw was entirely free from Teeth at Nine Years of Age.* By SAMUEL PARKER, Birmingham.

THE following interesting case came under my notice, at the Middlesex Hospital, in June, 1854:

A boy, nine years of age, came from the country, attended by a young woman, (from whom I learnt the history of the case,) for the purpose of having a temporary lower molar extracted.

The permanent teeth already cut in the lower jaw were regular and well formed; but the upper jaw, as represented in the engraving, was entirely free from temporary or permanent teeth.

The jaw was much smaller than is usual at such an age, and measured from A to A 1⅝ inch, and from *B* to B 1⅜ inch.

The average size, according to models in my collection, being 2 inches from A. A, and 1⅝ inch from B to B.

I was informed that the temporary teeth had all been removed at different times, on account of pain; and that the jaw had been without teeth for nearly four years.

At the time I saw the boy, one tooth had just pierced the gum, which proved upon examination, to be the bicuspid of the left side; but there was not the slightest appearance of any others coming.

Many cases are on record in which one or two permanent teeth have been wanting; but the case just quoted appears to be the first in which *all* were absent. The absence of one or two permanent teeth may be accounted for by the non-appearance of the fungous or carneous substance (observed by Lafrogur, and more carefully investigated by Detabane,) behind the root of the temporary tooth, and supposed to be placed there for the purpose of carrying out the process of absorption. "If this fleshy tubercle," says Detabane, "fails to be developed, or is destroyed by injurious operation, the permanent tooth frequently remains in the socket, and never makes its appearance."

How are we to know in the living subject, whether this substance is developed or not? And are we to suppose that by the absence of all the permanent teeth, as in this case, the fleshy tubercle of Detabane's had not been developed, or had been destroyed by injurious operations ?

There can be but little doubt that the early extraction of the temporary teeth had been the cause of a greater part of this mischief; as such extractions are well known to be the cause of a great many dental irregularities. I have just seen in the *Dental News Letter*, for Janury, 1857, a case of a toothless person. "A Mr. W—— recently died, at an advanced age, who never had a tooth. His gums were very hard, and much resembled those of a catfish." A model of the first case is to be seen in the Museum of the College of Dentists of England.

*Lond. Quar. Jour. Dent. Science.*

## Figures and Tables

**Figure f1:**